# Associations of the -238(G/A) and -308(G/A) TNF-α Promoter Polymorphisms and *TNF-α* Serum Levels with the Susceptibility to Gastric Precancerous Lesions and Gastric Cancer Related to *Helicobacter pylori *Infection in a Moroccan Population

**DOI:** 10.31557/APJCP.2020.21.6.1623

**Published:** 2020-06

**Authors:** Ghizlane Bounder, Mohamed Reda Jouimyi, Hasna Boura, Eliette Touati, Valerie Michel, Wafaa Badre, Hassan Jouhadi, Maria Kadi, Meriem Eljihad, Hakima Benomar, Anass Kettani, Halima Lebrazi, Fatima Maachi

**Affiliations:** 1 *Helicobacter Pylori and Gastric Pathologies Laboratory, Institut Pasteur du Maroc, Casablanca, Morocco. *; 2 *Biology and Health Laboratory, Faculty of Sciences Ben M’sik, University Hassan II, Casablanca, Morocco. *; 3 *Pathogenesis of Helicobacter Laboratory, Institut Pasteur, Paris, France. *; 4 *Gastroenterology Department, Ibn Rochd University Hospital Center, Casablanca, Morocco. *; 5 *Department of Radiotherapy Oncology, Ibn Rochd University Hospital Center, Casablanca, Morocco. *; 6 *Histo-Cytopathology Laboratory, Institut Pasteur du Maroc, Casablanca, Morocco. *

**Keywords:** Gastric carcinogenesis, TNF-α polymorphisms, TNF-α serum levels, Helicobacter pylori

## Abstract

**Objective:**

*Helicobacter pylori (H. pylori)* induces the production of tumor necrosis factor-alpha (TNF-α), which is closely related to a gastric epithelial injury. *TNF-α* gene polymorphism and *TNF-α* serum levels are associated with various malignant conditions. Identification of the ideal marker for gastric cancer (GC) is still the leading aim of several trials. Physio-pathological considerations of GC led us to investigate the association of two *TNF-α* promoter polymorphisms (-308G>A and -238G>A), and TNF-α serum levels with the susceptibility to gastric precancerous (PL) and GC.

**Methods:**

Patients suffering from gastric lesions (65 chronic gastritis, 50 PL, 40 GC) related to *H. pylori* ‎infection , and 63 healthy controls (HC) were involved in this study. Individuals are genotyped by *TNF-α* gene promoter sequencing and TNF-α serum levels are measured by ELISA quantitative method.

**Results:**

Regarding TNF-α-308 G/A locus, we noticed higher risk for GC (OR=4.3, CI 1.5-11.9, p-value=0.005) and PL (OR=3.4, CI 1.2-9.2, p-value=0.01) for individuals with AA/GA genotypes compared to GG genotype. Concerning TNF-α-238 G/A locus, we noticed higher risk for GC (OR=5.9, CI 1.2-27.5, p-value=0.01) and PL (OR=4.8, CI 1.3-18, p-value=0.01) for individuals with GG genotype compared to AA/GA genotypes. We noticed that TNF-α serum levels have been increased together with gastric lesions severity. Moreover, TNF-α-308 and TNF-α-238 A alleles seemed to, respectively, upregulate and downregulate TNF-α serum levels.

**Conclusion:**

The TNF-α -308 A allele has a promotive effect for GC progression, whereas the TNF-α -238 A allele has a protective function against GC progression. High levels of TNF-α seemed to be associated with the aggressiveness of gastric lesions. *TNF-α *gene polymorphisms and TNF-α serum levels might be helpful to select those patients who are at high risk for GC.

## Introduction

Gastric cancer (GC) is one of the most aggressive neoplasms worldwide. It is responsible for over 1,000,000 new cases in 2018 and an estimated 783,000 deaths, making it the fifth most frequently diagnosed cancer and the third leading cause of cancer death (Bray et al., 2018). The process of gastric carcinogenesis is triggered by *Helicobacter pylori (H. pylori)*-driven inflammation, which leads to chronic gastritis which progresses to atrophy, and intestinal metaplasia. The metaplastic epithelium can undergo from other genomic and phenotypic disorganizations (dysplasia), which can then progress to invasive neoplasia (Koulis et al., 2019) .

*H. pylori* infection induces chronic inflammation in gastric mucosa which is a critical step in gastric carcinogenesis (Bockerstett and DiPaolo, 2017). Tumor Necrosis Factor-alpha (TNF-α) is one of the major cytokines associated with *H. pylori* infection and it is closely related to gastric epithelial injury (Rivalino et al., 2018; Yamamoto et al., 2004). TNF-α is a multifunctional cytokine that elicits various biological functions such as immune homeostasis, inflammatory reaction, and cell surveillance (Martínez-Reza et al., 2017). In cancer pathologies, TNF-α plays a paradoxical role. On one hand, this cytokine can act as a tumor suppressor through vascular destruction, tumor necrosis, and immunostimulatory effects. On the other hand, TNF-α can act as a tumor promoter by inducing cellular transformation, survival, proliferation, invasion, angiogenesis, and metastasis (Josephs et al., 2018; Martínez-Reza et al., 2017; Wajant, 2009; Wang and Lin, 2008) . 

The gene coding for TNF-α is located in the short arm of chromosome 6 (6p21) within the highly polymorphic region of the major histocompatibility complex. Its location and strict linkage disequilibrium present between some alleles of HLA-DR class II and HLA-B class I genes have allowed to hypothesize associations between TNF-α alleles and some diseases (Cereda et al., 2012). The promoter region of the *TNF-α* gene (approximately 1,000 bp) contains a very large number of regulatory elements that affect the transcription of the gene (Cereda et al., 2012). The expression of TNF-α is tightly regulated, in part, at the transcriptional level. Several polymorphisms within TNF-α promoter region (-308, -857, -863, −851, −575, −376, −244, -238, -1031) have been reported to influence the expression of *TNF-α* gene and diseases susceptibility (Banday et al., 2016; Cereda et al., 2012) . 

The *TNF-α* gene encodes for a transmembrane protein (tmTNF-α) consisting of 233 amino acids (26 KDa) that is released in a soluble form (sTNF-α) of 157 amino acids (17 KDa) by TNF-converting enzyme-mediated cleavage (Martínez-Reza et al., 2017). The different TNF-α isoforms can have different effects on inflammatory responses and tumor development. Based on several studies, it seems that tmTNF-α reduces the severity of inflammatory reactions and tumor growth, while sTNF-α stimulates those processes (Ardestani et al., 2013; Horiuchi et al., 2010; Uysal et al., 2018; Yamamoto et al., 2004; Yu et al., 2013). 

A high level of TNF-α is associated with aggressive behavior and poor prognosis in many malignant cancers (Gao et al., 2017; Rossi et al., 2018). 

The sTNF-α is detected at low doses in the serum of healthy subjects, while high levels are detected in patients suffering from cancer and inflammatory diseases (Erturk et al., 2016; Mlak et al., 2020; Morningstar-Wright, 2018; Mourtzikou et al., 2014; Stanilov et al., 2014). Decreases in TNF-α serum levels in patients receiving chemotherapy have been reported by many studies (Berberoglu et al., 2004). This suggests that the serum levels of sTNF-α could be an indicator of the response to chemotherapy and tumor prognosis.

In most patients, GC is diagnosed in an advanced stage, which explains the poor prognosis and the high mortality rates. GC develops via several sequential lesions, which representing an opportunity for early detection and intervention to prevent the progression of precancerous lesions (PL) towards the GC. The aim of the present study is to evaluate the potential of TNF-α genotyping (TNF-α -308 G/A, TNF-α -238 G/A) and TNF-α serum levels as predictive markers in GC. 

## Materials and Methods

*Clinical and pathological characteristics of the study population*

Our study focused on patients suffering from gastric pathologies related to *H. pylori* infection collected from Gastroenterology and Oncology departments at the IBN ROCHD University Hospital Center. Patients having received previous treatment for *H. pylori* eradication, proton pump inhibitors, anti-inflammatory medicines, chemotherapy or radiotherapy treatment, patients suffering from any other infection, inflammatory disorders, cancers other than distal gastric adenocarcinoma, were excluded from the study. Healthy asymptomatic subjects, with no history of gastrointestinal illnesses or regular use of any gastrointestinal and anti-inflammatory medicines, were recruited from the Regional Transfusion Center of Casablanca .Three biopsies (1 antrum, 1 fundus, 1 lesser curvature) were sampled from patients admitted for endoscopy. Blood samples have been sampled from all participants. 

Clinical information about the demographic characteristics of the participants including age, sex, place of birth, drug consumption (antibiotics, PPIs, anti-inflammatory drugs), smoking and alcohol habits, were collected using a structured questionnaire. All participants were informed about their inclusion in the study and agreed to it in writing form. The study protocol has been performed in accordance with the ethical standards of Helsinki and was approved by the ethical committee of Pasteur Institute of Morocco. 

The biopsies were fixed in 10% formalin and taken to Histo-Cytopathology laboratory in Pasteur Institute of Morocco, where they were embedded in paraffin blocks, sectioned and stained in hematoxylin and eosin for conventional histopathology examination of gastric ‎mucosal lesions and *H. pylori* infection. 

The quantitative anti-H.Pylori IgG ELISA method was performed to detect *H. pylori* infection in the healthy controls subjects, using a commercially available kit (Euroimmun, D-23560 Lubeck (Deutschland)). A cut-off value of antibody concentration ≥ 20 relative units (RU)/ml was considered as positive.

*DNA extraction and quantification* *‎*

Genomic DNA was extracted from blood taken on EDTA tube using the commercially available kit (PureLink™ Genomic DNA Mini Kit). The quantity, quality, and purity of DNA were checked by 0.8 % agarose gel electrophoresis and also by the ratios of Optical Density at 260/280 nm and 260/230 nm using a spectrophotometer (NanoVue plus). Then it was stored at −20°C until use.

*Genotyping of TNF-α -308 /-238 polymorphisms*

Individuals are genotyped for *TNF-α* gene promoter at position -308 (G/A) and -238 (G/A) by using sequencing.

Firstly, the *TNF-α* gene (266 bp) was amplified by PCR. The reaction was carried out in a total volume of 20 µL, consisting of 200 ng of genomic DNA, 0.5 µM of primers (Essadik et al., 2015), 1 mM de dNTPs, 3 mM MgCl_2_ and 0.5 unit of Taq polymerase (BIOLINE). Thermocycling conditions of PCR were as follows: initial denaturation at 95°C for 1min, followed by 35 cycles of 95°C for 15 seconds, 57°C for 15 seconds, 72°C for 30 seconds, and a final extension at 72°C for 7 min. The PCR products were ‎analyzed by 1.5% agarose gel electrophoresis, purified using ExoSap (EX’S-Pure, Nimagen) in a final volume of 7 µL, consisting of 2 µl ExoSAP enzyme and 5µl of PCR product, and incubated at 37°C for 4min and 90°C for 1min. Then, The DNA sequencing conditions was carried out on the basis of the method described by Essadik (2005) by using BigDyeR Terminator v3.1 Cycle Sequencing Kit (AppliedBiosystems). The DNA sequences were read by an Applied Biosystems 377 DNA sequencer ‎and analyzed using the BIOEDITE software package. ‎

*Measurement of TNF-α serum Levels*

Blood samples were collected in a serum tube from each patient before anesthesia and gastroscopy examination and conserved in an icebox during transport. The serum was separated within 1 h of blood collection after spinning for 15 min at 1,500 g. Serum samples were stored at -80 °C in aliquots of 500 µL and thawed just before testing. Serum levels of TNF-α were measured using the Human TNF-α ELISA kit from R and D Systems (DuoSet ELISA, USA) and it was used according to the manufacturer’s instructions. Total TNF-α concentrations in samples were expressed as pg/ml. 

*Statistical analysis*

The results of this study were statistically analyzed using R software version 1.1.456. The descriptive data are presented in terms of frequencies. The differences between the groups were analyzed with the chi-square or Fisher’s exact test for the categorical variables, Wilcoxon test for the qualitative (2 classes) and numeric variables, and the Kruskal Wallis test for the qualitative (3 classes and more) and numeric variables. The differences were considered significant at p-value <0.05. 

## Results

*Clinical and pathological characteristics of the study population*

In total, 218 participants with *H. pylori*-positive status were selected in the present study and ‎divided into two categories. The first category includes 63 asymptomatic healthy controls subjects (HC). The second category includes 155 patients with various gastric lesions related to *H. pylori* infection: 65 with chronic gastritis, 50 with PL (30 with atrophic gastritis, 20 with ‎intestinal metaplasia) and 40 with gastric adenocarcinoma, were investigated. Male/Female ‎ratio in the case group and the control group was, respectively, 73/82 and 34/29. The mean age of HC, chronic gastritis, PL and GC was, respectively, 43 ±8, 45±16, 53±14, 56±14 years. 

*Genotyping of TNF-α -308 /-238 polymorphisms*

Regarding TNF-α promoter -308 G/A locus, the frequency of A allele is 8% in HC and has shown an increase with the severity of gastric lesions, with the following order: 10.8% in chronic gastritis, 21% in PL and 27.5% in GC. Besides, the frequency of GA genotype ‎was observed higher in patients with chronic gastritis, PL and GC (12.3%, 18%, and 15% respectively) compared with the HC group (6.3%). Whereas the AA genotype was more occurred among patients with PL and GC (12% and 20% respectively) compared to patients with chronic gastritis and HC (4.6% and 4.8% respectively) (Table 1). Significant alterations in genotypes and alleles distribution of TNF-α -308 G/A locus between patients with GC (p-value=0.01, p-value=0.0001 respectively), PL (p-value=0.04, p-value=0.004 respectively), and control group were noticed. Moreover, a higher risk for GC (OR= 4.3, CI 1.5-11.9, p-value=0.005) and PL (OR= 3.4, CI 1.2-9.2, p-value= 0.016) was seen for individuals carrying TNF-α -308 AA/GA genotypes against those with TNF-α -308 GG genotype. Whereas no significant differences between chronic gastritis and control group were identified. 

Regarding TNF-α promoter -238 G/A locus, the frequency of A allele is 17% in HC and it was observed in decline together with the increase of gastric lesions severity, in the following order: 10.7% in chronic gastritis, 4% in PL and 3.5% in GC. Besides, the genotypes GA and AA has occurred more among HC (14.3% and 9.5% respectively) and patients with chronic gastritis (12.3% ‎and 4.6% respectively) compared to patients suffering from PL (4% and 2% respectively) and GC (2.5% and 2.5% respectively). The genotypes and alleles distribution of TNF-α -238 G/A locus were statistically different between HC and patients with PL and GC (p-value=0.04) (Table 1). Besides, a higher risk for GC (OR= 5.9, CI 1.2-27.5, p-value=0.01) and PL (OR= 4.8, CI 1.3-18, p-value=0.01) were noticed for individuals with ‎ TNF-α -238 GG genotype against AA/GA genotypes. Whereas no significant differences between chronic gastritis and control group were identified. 

*Measurement of TNF-α serum Levels*

The relationship between serum levels of circulating TNF-α and the severity of gastric lesions was investigated. The distribution of TNF-α levels in each group of patients and controls is shown in Table 2.

The serum levels of circulating TNF-α were observed to be increased together with the severity of gastric lesions. TNF-α serum levels in the GC group were higher than those recorded in patients with PL and patients with chronic gastritis compared to HC. TNF-α serum levels mean were as follows 36 pg/ml in HC, 50.7 pg/ml in chronic gastritis, 55 pg/ml in PL, and 116.6 pg/ml in GC. A disparity in the TNF-α serum levels between patients and HC, and between GC and the other gastric lesions cases were noted, and it was statistically meaningful (p-value < 0.05). 

*Association between TNF-α -308 /-238 polymorphisms and TNF-α serum levels*

The impact of the TNF-α -308 G/A and -238 G/A polymorphisms on its serum levels were assessed. Table 3 shows a noteworthy difference in TNF-α levels between different genotypes of the two loci.

Regarding TNF-α -308 G/A locus, the TNF-α serum levels were observed to be increased with possession of the A allele. The mean level of TNF-α was 44.6 pg/ml within individuals harboring GG genotype, 75.1 pg/ml within GA, and 205.4 pg/ml within those harboring AA. The difference was statistically significant between TNF-α -308 polymorphism and TNF-α serum levels (p-value = 7.1e-06). 

Concerning TNF-α -238 G/A locus, the TNF-α serum levels were observed to be decreased with possession of the A allele. The mean level was 54.4 pg/ml, 36.5 pg/ml and 34.1 pg/ml within individuals harboring GG, GA and AA genotypes, respectively. The difference was statistically significant between TNF-α -238 polymorphism and TNF-α serum levels (p-value=0.0003). 

**Table 1:** TNFα Promoter Polymorphisms at Positions -308 and -238 in Patients with Gastric Lesions and Healthy Controls

	HC	CG	PL	GC
	N (%)	N (%)	N (%)	N (%)
TNFα -308 G/A genotypes				
GG	56 (88.9)	54 (83.1)	35 (70)	26 (65)
GA	4 (6.3)	8 (12.3)	9 (18)	6 (15)
AA	3 (4.8)	3 (4.6)	6 (12)	8 (20)
* P*-value*		0.5	0.044	0.012
OR; [95%CI]; *P*-value (AA + GA vs. GG)**		1.62,[0.6-4.5];0.44	3.4;[1.2-9.2];0.016	4.3;[1.5-11.9];0.005
TNFα -308 G/A alleles				
G	116 (92)	116 (89.2)	79 (79)	58 (72.5)
A	10 (8)	14 (10.8)	21 (21)	22 (27.5)
* P*-value*		0.46	0.004	0.0001
OR;[95%CI]; *P*-value (A vs. G)**		1.4;[0.6-3.2];0.52	3.08; [1.3-6.9];0.006	4.4; [1.95-9.9]; 3e-04
TNFα -238 G/A genotypes				
GG	48 (76.2)	54 (83.1)	47 (94)	38 (95)
GA	9 (14.3)	8 (12.3)	2 (4)	1 (2.5)
AA	6 (9.5)	3 (4.6)	1 (2)	1 (2.5)
* P*-value*		0.54	0.04	0.04
OR; 95% CI; *P*-value** (GG vs. AA + GA)		1.5;[0.64-3.6]; 0.38	4.8; [1.3-18];0.01	5.9;[1.2-27.5]; 0.01
TNFα -238 G/A alleles				
G	105 (83)	116 (89.3)	96 (96)	77 (96.5)
A	21 (17)	14 (10.7)	4 (4)	3 (3.5)
* P*-value*		0.17	0.002	0.004
OR; 95%C I; *P*-value** (G vs. A)		1.6; [0.8-3.4]; 0.2	4.8; [1.5-14.4]; 0.002	5.1;[1.4-17.8]; 0.006

HC, Healthy controls; CG, chronic gastritis; PL, precancerous lesions; GC, gastric cancer. *p-value was calculated using the Chisq test/Fisher test. ** twoby2

**Table 2:** Association between Serum Levels of TNFα and Gastric Lesions Severity

TNFα (pg/mL)	HC	CG	PL	GC
Range	6-77.7	22-100	11-108	31- 289.7
Mean	36	50.7	55	116.6
SD	15.6	19.6	27.5	91.8
p-value significance intra-groups *	8.00e-09			
p-value significance inter-groups **				
Case vs controls		0.001	0.0004	2.20E-08
Case vs chronic gastritis		-	0.2	0.00002
Case vs precancerous lesions		-	-	0.001

HC, Healthy controls; CG, chronic gastritis; PL, precancerous lesions; GC, gastric cancer; *, Kruskal-Wallis test; **, Wilcox test.

**Table 3:** Association between TNFα -308 and -238 G/A Locus Genotypes and TNFα Serum Levels

	TNFα -308 G/A Genotypes			TNFα -238 G/A Genotypes		
TNFα (pg/mL)	GG	GA	AA	GG	GA	AA
Range	11-85	23-183.2	6-595	6-113.2	11-64.2	6-55
Mean	44.6	75.1	205.4	54.4	36.5	34.1
SD	17.9	48.6	197.1	28.4	13.8	16
p-value significance intra-groups *		7.10e-06		0.0003		
p-value significance inter-groups **						
Case vs GG		0.001	0.00007		0.0008	0.01
Case vs GA			0.03			0.92

*, Kruskal-Wallis test; **, Wilcox test.

## Discussion

Identification of the ideal marker for GC is still the leading aim of several trials. Physio-pathological considerations of gastric carcinogenesis led us to evaluate the potential of TNF-α genotyping (TNF-α -308 G/A, TNF-α -238 G/A) and TNF-α serum levels as predictive markers of GC.

In our population, the evaluation of the association between TNF-α polymorphisms and the susceptibility to GC has revealed a noteworthy link.

Regarding TNF-α -308 G/A locus, the frequency of the TNF-α -308 A allele was observed to increase with gastric lesions severity. Notable disparity in TNF-α -308 genotypes frequencies is observed between patients and HC. Indeed, the GA genotype was more often detected in patients with chronic gastritis, PL and GC compared to HC. Whereas the AA genotype was mostly detected in patients suffering from PL and GC. Our results are consolidated with several studies reported the association of TNF-α -308 G/A polymorphism with increased susceptibility to various cancers, including hepatocellular carcinoma, prostate cancer, oral cancer, lung cancer, cervical cancer, oesophageal cancer, breast cancer and colorectal cancer (Babapour et al., 2019; Banday et al., 2016; Pan et al., 2012; Wu et al., 2018). In gastric diseases, TNF-α-308 G/A is associated with a high risk of chronic atrophic gastritis, intestinal metaplasia and GC (Du and Gao, 2017; Karaman et al., 2014; Machado et al., 2003). 

Regarding TNF-α -238 G/A polymorphism, the frequency of TNF-α -238 A allele and TNF-α-238 GA/AA genotypes were observed lower in patients with PL and GC compared to chronic gastritis and HC. According to other reports, TNF-α -238 promoter polymorphism seems to be associated with attenuated susceptibility to various cancers, including gastric carcinoma, uterine cervical carcinoma, colorectal carcinoma, renal cell carcinoma and lung cancer (Jang et al., 2001; Pan et al., 2012; Shih et al., 2006). 

The relationship between serum levels of circulating TNF-α and gastric carcinogenesis was investigated in this study. A significant disparity of TNF-α serum levels between cases and controls was noticed. We remarked that TNF-α serum levels increase among patients with chronic gastritis, with PL and mainly in those suffering from GC.

High levels of TNF-α have been described in several studies among patients suffering from cancer, including gastric carcinoma, colorectal carcinoma (Erturk et al., 2016; Stanilov et al., 2014), hepatocellular carcinoma (Wang et al., 2003), head and neck cancer (Mlak et al., 2020). Furthermore, a high level of TNF-α is associated with aggressive behaviour and poor prognosis in many malignant cancers (Gao et al., 2017; Rossi et al.,2018). TNF-α plays a critical role in defending against *H. pylori* infection and in contributing to gastric inflammation (Morningstar-Wright, 2018; Thalmaier et al., 2002). TNF-α is implicated in intestinal metaplasia and hyperplastic gastric tumors (Oshima et al., 2005; Senthilkumar et al., 2011), through the stimulation of some proteins expression required in the development of intestinal cell, such as CDX1 and CDX2, intestinal mucin MUC2 and MUC4 (Ahn et al., 2005; Coskun et al., 2011; Mejías-Luque et al., 2010). These findings support the elevation of TNF-α serum levels in our patients suffering from chronic gastritis, PL and GC.

The regulation of TNF-α expression is in part genetically determined. Our finding suggests that TNF-α-308 A allele may be related to an increase in TNF-α serum levels. This result agreed with previous studies reported the positive impact of this polymorphism on TNF-α production (Abraham and Kroeger, 1999; Prasad et al., 2010; Wilson et al., 1997). Regarding TNF-α-238 G/A locus, we found TNF-α-238 A allele associated with a decrease in TNF-α serum levels. This finding is supported by other studies reported the downregulation of TNF-α production by TNF-α-238 polymorphism (D’Alfonso and Richiardi, 1994; Kaluza et al., 2000). 

TNF-α -308 A allele was more recorded in the GC group and was linked to high TNF-α levels. In contrast, TNF-α -238 A allele was less detected in the GC group and was associated with low TNF-α levels, we can suggest that the elevation of circulating TNF-α is implicated in the promotion of GC and not in its regression as a double act of this cytokine vis-a-vis malignant and precancerous cell. It seems that tmTNF-α reduces the severity of inflammatory reactions and tumor growth, ‎while sTNF-α stimulates those processes (Ardestani et al., 2013; Horiuchi et al., 2010; Uysal et al., 2018; Yamamoto et al., 2004; Yu et al., 2013).

In conclusion, TNF-α -308 A and TNF-α -238 A allele seem to have a promotive and protective effect respectively on GC development. Higher TNF-α serum levels appear associated with gastric carcinogenesis. Furthermore, TNF-α -308 A and TNF-α -238 A allele seem to upregulate and downregulate TNF-α ‎serum levels, respectively‎. Further studies including a large population and diverse ethnicities are needed to illumine the association of TNF-α-308 G/A and TNF-α-238 G/A polymorphisms and TNF-α serum levels with GC. QRT-PCR and Western blotting assays are recommended to confirm the relationship between TNF-α expression and TNF-α polymorphisms . 
